# Mega‐map of systematic reviews and evidence and gap maps on the interventions to improve child well‐being in low‐ and middle‐income countries

**DOI:** 10.1002/cl2.1116

**Published:** 2020-10-28

**Authors:** Ashrita Saran, Howard White, Kerry Albright, Jill Adona

**Affiliations:** ^1^ Campbell South Asia Vasant Kunj Delhi India; ^2^ Campbell Collaboration Vasant Kunj Delhi India; ^3^ UNICEF Office of Research‐Innocenti Florence Italy; ^4^ Philippines Institute of Development Studies Manila Philippines

## Abstract

**Background:**

Despite a considerable reduction in child mortality, nearly six million children under the age of five die each year. Millions more are poorly nourished and in many parts of the world, the quality of education remains poor. Children are at risk from multiple violations of their rights, including child labour, early marriage, and sexual exploitation. Research plays a crucial role in helping to close the remaining gaps in child well‐being, yet the global evidence base for interventions to meet these challenges is mostly weak, scattered and often unusable by policymakers and practitioners. This mega‐map encourages the generation and use of rigorous evidence on effective ways to improve child well‐being for policy and programming.

**Objectives:**

The aim of this mega‐map is to identify, map and provide an overview of the existing evidence synthesis on the interventions aimed at improving child well‐being in low‐ and middle‐income countries (LMICs).

**Methods:**

Campbell evidence and gap maps (EGMs) are based on a review of existing mapping standards (Saran & White, 2018) which drew in particular of the approach developed by 3ie (Snilstveit, Vojtkova, Bhavsar, & Gaarder, 2013). As defined in the Campbell EGM guidance paper; “Mega‐map is a map of evidence synthesis, that is, systematic reviews, and does not include primary studies” (Campbell Collaboration, 2020). The mega‐map on child well‐being includes studies with participants aged 0–18 years, conducted in LMICs, and published from year 2000 onwards. The search followed strict inclusion criteria for interventions and outcomes in the domains of health, education, social work and welfare, social protection, environmental health, water supply and sanitation (WASH) and governance. Critical appraisal of included systematic reviews was conducted using “A Measurement Tool to Assess Systematic Reviews”‐AMSTAR‐2 rating scale (Shea, et al., 2017).

**Results:**

We identified 333 systematic reviews and 23 EGMs. The number of studies being published has increased year‐on‐year since 2000. However, the distribution of studies across World Bank regions, intervention and outcome categories are uneven. Most systematic reviews examine interventions pertaining to traditional areas of health and education. Systematic reviews in these traditional areas are also the most funded. There is limited evidence in social work and social protection. About 69% (231) of the reviews are assessed to be of low and medium quality. There are evidence gaps with respect to key vulnerable populations, including children with disabilities and those who belong to minority groups.

**Conclusion:**

Although an increasing number of systematic reviews addressing child well‐being topics are being published, some clear gaps in the evidence remain in terms of quality of reviews and some interventions and outcome areas. The clear gap is the small number of reviews focusing explicitly on either equity or programmes for disadvantaged groups and those who are discriminated against.

**Abbreviations**
3ie: International Impact Evaluation Initiative
AIDS: acquired immunodeficiency syndrome
CRC: (United Nations) Convention on the Rights of the Child
DID: difference in difference
EGM: evidence and gap map
GDP: gross domestic product
HIV: human immunodeficiency virus
INSPIRE: Implementation and enforcement of laws, Norms and values, safe environments, Parent and Caregiver support, Income and economic strengthening, response and support services, Education and life skills
IV: instrumental variables
LMIC: low and middle income country
PSM: propensity score matching
RCT: randomised controlled trial
RDD: regression discontinuity design
SDG: sustainable development goals
UN: United Nations
UNDP: United Nations Development Programme
UNICEF: United Nations Children's Fund
WHO: World Health Organisation

## 1 PLAIN LANGUAGE SUMMARY

### 1.1 Mega‐map of studies shows large but unevenly distributed research base on child well‐being in low‐ and middle‐income countries (LMICs)

Research plays a crucial role in helping to close gaps in child well‐being in LMICs, yet the global evidence base for interventions to meet these challenges is mostly weak, scattered and often unusable. There is now a mega‐map that encourages the generation and use of rigorous evidence on effective ways to improve child well‐being for policy and programming.

#### 1.1.1 What is the child well‐being mega‐map?

This child well‐being mega‐map shows evidence synthesis studies—systematic reviews and evidence and gap maps (EGMs)—that include studies on the effectiveness of interventions to improve child well‐being in LMICs published in English.

The mega‐map provides a visual and interactive display of completed and on‐going studies structured around a framework: a matrix of interventions and outcomes primarily based on the strategic plan of the United Nations Children's Fund (UNICEF, 2018). This includes seven intervention categories; early child development, health and nutrition, education, social work and welfare, social protection, environmental health including water, sanitation and hygiene (WASH), and governance.

**What is the aim of this mega‐map?**

This child well‐being mega‐map provides a visual and interactive display of evidence synthesis studies—systematic reviews and evidence and gap maps—on the effectiveness of interventions to improve child well‐being in low‐ and middle‐income countries.

#### 1.1.2 What studies are included?

The mega‐map includes 356 studies that were published from 2000 to 2018, with interventions to improve child well‐being of those aged 18 years and under.

Systematic reviews are included if the inclusion criteria in the reviews mentioned studies from LMICs irrespective of whether studies from LMICs were actually identified. Reviews with multiple populations are included in the map if they had an intervention for child well‐being.

#### 1.1.3 What are the main findings of this map?

This mega‐map includes 333 systematic reviews and 23 EGMs. Of the included studies, there is substantial evidence in traditional areas: health and nutrition (234) is the most commonly studied intervention, followed by early child development (194). Education (83) and social protection (62) are fairly well‐represented. However, there is limited evidence synthesis in nontraditional areas: social work and welfare (44), environmental health including WASH (41) and governance (13).

The most commonly studied outcomes are health (259) and healthy development (215). Very few studies measured economic impact (42) and risk factor reduction (55) of these interventions.

In terms of quality, 26% of the reviews were identified to be of low quality.

The mega‐map reveals a lack of evidence on vulnerable children: children with disabilities and children in conflict‐affected settings.

Systematic reviews were found to be almost equally concentrated across all regions with sub‐Saharan Africa (292) and South Asia (268) having the highest number of reviews. These were closely followed by Latin America and Caribbean (245), East Asia and Pacific (243) and Middle East and North Africa (234).

#### 1.1.4 What do the findings of the map mean?

Whilst the evidence base is relatively large, it is unevenly distributed across intervention categories. There is a need for more studies which are explicitly focused on programmes for vulnerable children. There are limited systematic reviews focusing on gender and equity issues in child well‐being. Very few studies were identified assessing programme cost of the interventions.

It would be useful for global and country partners to work together to achieve consensus on priority areas for evidence synthesis. They should also adopt a coordinated approach to undertaking and updating maps and reviews across priority areas.

#### 1.1.5 How up‐to‐date is the mega‐map?

The authors searched for studies published up to December 2018.

## 2 EXECUTIVE SUMMARY

### 2.1 Background

Well‐being can be defined as the realisation of children's rights and the fulfilment of the opportunity for every child to be all she or he can be in the light of a child's abilities, potential and skills. The degree to which this is achieved can be measured in terms of positive child outcomes, whereas negative outcomes and deprivation point to the neglect of children's rights (Bradshaw, Hoelscher, & Richardson, 2007). Monitoring, protecting and promoting well‐being is central to realisation of children's rights, as set out in the UN Convention on the Rights of the Child, (UNICEF, Convention on the Rights of the Child, 1989)

According to UNICEF's “State of the World's Children” report (Keeley & Little, 2017), there are 2.2 billion people aged under the age 18 and under and the majority of these are living in poverty. Evidence from countries across the globe shows that children who grow up in poverty are more likely to experience poor health, fewer opportunities to access good quality education and to be low paid or unemployed in the future (Keeley & Little, 2017). Children living in extremely poor households are unevenly concentrated in certain parts of the developing world. Sub‐Saharan Africa has the highest rates of children living in extreme poverty at just under 49%, and South Asia has the second highest share at nearly 36% (UNICEF and the World Bank Group, 2016).

Research plays a crucial role in helping to close the remaining gaps in child well‐being, yet the global evidence base for effective interventions to meet these challenges is mostly weak, scattered and often unusable by policymakers and practitioners. This short report summarises findings from an EGM undertaken by Campbell South Asia with support from the UNICEF Office of Research‐ Innocenti. We would like to acknowledge and thank Bill and Melinda Gates Foundation for their funding support to make it a living map, updated annually over the lifetime of UNICEF's Strategic Plan 2018–2021.

### 2.2 Objectives

The mega‐map has two main objectives:

Identify, map and describe existing systematic reviews and EGMs on the effectiveness of interventions that aim to improve well‐being of children in low and middle income countries.

Identify existing gaps in terms of methodology, interventions, outcomes, geography and population subgroups to help researchers and policy makers with evidence‐informed research and funding decisions.

### 2.3 What is an EGM?

An EGM is a presentation of the available, relevant evidence for a particular sector. The map is a table or matrix which provides a visual presentation of the evidence. In the present map, the rows are intervention categories and the columns are indicator (outcome) categories. This mega‐map provides an overview of all available evidence (systematic reviews and EGMs) till December 2018 on the key outcome domains and interventions aimed at improving child and adolescent well‐being in LMIC countries using an intervention‐outcome framework.

### 2.4 Method

The mega‐map was developed in stages:

Framework construction:

The first step was to decide the scope of the mega‐map. Specifically, it involved identifying the primary dimensions of the map, that is, interventions and outcomes. We started developing the draft framework by reviewing UNICEF strategic plan documents (UNICEF, 2018). We also reviewed key documents by major funders in this sector such as WHO, UNDP, Department for International Development (DFID) and the World Bank. An expert group comprised of international experts in the field was formed. In consultation with the expert group, intervention and outcome categories were reviewed and modified and the scope was set. The map was discussed with stakeholders in two consultation meetings and the framework further refined.

Search Methodology:

Step 1: Included a search of relevant systematic reviews and EGMs from the 3ie database. This initial map included 99 studies.

Stage 2: A full database search was performed. The search was carried out on (a) Systematic Review databases, such as 3ie and Campbell Collaboration databases (full list is provided in the appendix) (b) Academic databases, such as OVID and Embase. This identified 302 relevant systematic reviews after the full database search.

Stage 3: (a) Additional websites were searched for grey literature, including those of bilateral and multilateral organisations, such as ILO and UNDP; websites of J‐PAL and ANU‐Lib. (b) Experts consulted, and (c) screened and coded studies received from key informants. This phase identified additional 54 studies for inclusion.

The search of the full academic databases and grey literature yielded 9,086 hits and 356 studies were included in the final map after screening and coding.

### 2.5 Selection criteria

The primary population of interest for this map is children and adolescents from LMICs (UNICEF, Convention on the Rights of the Child, 1989). Children irrespective of their sex in the age group of 18 years and under were included in the EGM. The age group is classified based on the age criteria stated as follows: infanthood (<3 years of age), childhood (3–10 years), adolescence (10–18 years). The population subgroup of interest includes: girls, orphans and vulnerable children, children with disabilities, children belonging to ethnic minorities, child sex workers, child brides, isolated children/street children, children with HIV/AIDS, migrants and children affected by conflict and humanitarian crises.

Systematic reviews are often global in scope. Systematic reviews were included if the “inclusion criteria” in the reviews was to include studies from LMICs unrelated to the fact that weather studies from LMICs were actually identified through search or included from screening. The screening process for mega‐map did not check whether the reviews included studies from LMICs. Studies with multiple populations are included in the map if they included an intervention for child well‐being.

### 2.6 Screening, data extraction and critical appraisal

Title and abstract screening and the evidence classification were undertaken by two independent reviewers, and any discrepancies were resolved by a third reviewer. The studies that passed on to full text stage were screened against the eligibility criterias by two independent reviewers and conflicts resolved by a third reviewer.

After screening, all studies were coded for a wide array of information and populated into the map. The studies were coded for bibliographic details, the interventions and outcomes and other relevant aspects such as population, region and countries. The coding was again carried out by two independent reviewers and conflicts were reconciled by a third reviewer. Critical appraisal appraisal of all included studies was performed by independent reviewers.

### 2.7 Results

The mega‐map includes 333 systematic reviews and 23 EGMs. A main finding from the map is that there is a substantial amount of evidence in the traditional areas such as early childhood development, health and nutrition and education. However, there is uneven distribution of evidence across region, quality of studies and intervention and outcome subcategories.

For example, though health and nutrition were the most populated areas of the map, some gaps were identified; management of severe acute malnutrition (8), agricultural intervention/biofortification (13), m‐health intervention for child health (16). In education, there is limited evidence around remedial education, nonformal education and scholarships and systemic intervention. In terms of quality, about 26% (58) of the systematic reviews were identified to be of low quality for health and nutrition. This was found to be similar for early childhood development (30%) and education (22%).

Striking gaps in evidence were identified in the nontraditional areas such as social protection and social welfare, with lack of evidence in many areas such as child trafficking, substance abuse prevention, interventions for child abuse, social insurance and labour market insurance. These were not just under‐researched but also under‐funded. The most funded systematic review areas have been health and nutrition and early childhood development over the years. The major funders of such reviews have been the DFID, World Health Organisation (WHO), Bill and Melinda Gates Foundation, National Institute of Health Research (NIHR) and UNICEF.

Health and learning outcomes were found to be the most studied as well. Few studies were identified assessing the impact of interventions on risk reduction, safety, equity, and economic outcomes.

UNICEF Strategic Goal 1 (Every child survives and thrives) and Goal 2 (Every child learns) are heavily populated. Goal 3 (Every child is protected from violence and exploitation) and Goal 4 (Every child lives in a clean and safe environment) are less populated and there is scant evidence in Goal area 5 (Every child has an equitable chance in life).

### 2.8 Conclusion

Although an increasing number of systematic reviews addressing child well‐being topics are being published, there is a need for more studies across some interventions and outcome areas such as social protection and social welfare. The clear gap is the small number of reviews focusing explicitly on either equity or programmes for disadvantaged groups and those who are discriminated against. There is a need for more studies which are explicitly focused on programmes for vulnerable children. There are limited systematic reviews focusing on gender and equity issues in child well‐being. Very few studies were identified assessing programme cost of the interventions.

It would be useful for global and country partners to work together to achieve consensus on priority areas for evidence synthesis and to adopt a coordinated approach to undertaking and updating maps and reviews across priority areas.

## 3 BACKGROUND

### 3.1 Introduction

#### 3.1.1 The state of child well‐being in LMICs

Child well‐being is a multidimensional and a holistic concept which provides a contextual understanding of a child in different domains such as health, material well‐being, education, conditions of housing and environment, and interpersonal relations (UNICEF, 2014). A decent level of child well‐being is underpinned by the UN convention of Rights of the Child which states that “The child, by reason of his physical and mental immaturity, needs special safeguards and care, including appropriate legal protection, before as well as after birth” (UNICEF, Convention on the Rights of the Child, 1989). But many children around the world still suffer deficiencies in many dimensions of well‐being.

#### 3.1.2 Aspects of shortfalls in child well‐being

According to UNICEF's “State of the World's Children” report (2017), there are 2.2 billion people aged under the age of 18 and the majority of these are living in poverty. Evidence from many countries shows that children who grow up in poverty are more likely to experience poor health, fewer opportunities to access good quality education and to be low paid or unemployed in the future (UNICEF 2017). One in three children (200 million globally) fails to reach their full physical, cognitive, psychological and/or socioemotional potential due to poverty, poor health and nutrition, insufficient care, stimulation and other risk factors related to early childhood development (Grantham‐McGregor, et al., 2007). It is estimated that globally, almost 385 million children are living in extreme poverty. Poverty, malnutrition, poor health, unstimulating home environments and violence against children are major risk factors, which detrimentally affect the cognitive, motor and social‐emotional development of children (Tran, Luchters, & Fisher, 2017). Children living in extremely poor households are unevenly concentrated in certain parts of the developing world. Sub‐Saharan Africa has the highest rates of children living in extreme poverty at just under 49%, and South Asia has the second highest share at nearly 36% (UNICEF, Ending extreme poverty: a focus on children. Sustainable Development Goals (SDGs) in Rich Countries, 2016). In 2017, the under‐five mortality rate in low‐income countries was 69 deaths per 1,000 live births—around 14 times the average rate in high‐income countries (five deaths per 1,000 live births). Preterm birth complications, acute respiratory infections, intrapartum‐related complications, congenital anomalies and diarrhoea are the main factors continuing causes of high numbers of under‐five deaths (WHO, 2019). Globally, millions of adolescents die or become sick from preventable causes such as road accidents, HIV, suicides and interpersonal violence (WHO, 2018). Further adding to the plight of children in developing countries, young girls and adolescent women are invariably subjected to various forms of harmful practices including child marriage and female genital mutilation. Education offers children a path to a promising future but about 264 million children and adolescents around the world fail to enter or complete school (UNESCO, 2017). These figures are alarming in 24 conflict‐affected regions with approximately 27 million children out of school (UNICEF, Goal Area 2 Every child learns‐Global annual report, 2018). They are thwarted by poverty, discrimination, armed conflict, emergencies and the effects of climate change. Often the family and environmental risk factors a child experiences are beyond their control. However, the effects of these factors can be moderated and this is where opportunities to promote children's well‐being and positive mental health lie.

#### 3.1.3 Consequences of shortfalls in child well‐being

Childhood deprivation cannot just take childhood from children, but also have long‐run consequences. Child undernutrition is associated with shorter adult height, less schooling, reduced economic productivity and lower offspring birthweight for women (Victoria et al., 2008). Lack of education is a major factor in households remaining poor (Baulch, 2011). Early marriage is bad for the health of the mother—with greater risk of dying during childbirth—and her offspring who are at greater risk of having low birth weight and of dying prematurely (Nour, 2006).

#### 3.1.4 Addressing shortfalls in child well‐being

The provision of services on health, education and safety to all children in the world irrespective of cast, creed, colour and ethnicity is a fundamental right enshrined in the UN Convention on the Rights of the Child (UNICEF, Convention on the Rights of the Child, 1989).

In recognition of the 2030 Agenda for Sustainable Development, children's rights and well‐being are acknowledged as important for long‐term sustainable development of children. Many SDGs are important reference points for the design of national development strategies for child well‐being including: End poverty (SDG1), End hunger, achieve food security and improved nutrition and promote sustainable agriculture (SDG2), health (SDG3), quality education (SDG4), reduce inequality between and within countries (SDG10). Despite this apparent focus on child well‐being and various international organisations working towards a common goal, striking gaps remain in achieving SDG's.

Research plays a crucial role in helping to close the remaining gaps in the global evidence base for effective interventions. SDG 17 targets 17.16 and 17.18 emphasise the increased need for investment in generating sound evidence to improve child well‐being intervention strategies. Though child well‐being interventions have been underway for decades; evidence on the effectiveness of these interventions is often scattered, the value is possibly underestimated and inclusion in national strategies and programmes is rare. Failure to effectively implement evidence‐informed interventions represents a key obstacle in the progress of child well‐being in many LMICs towards achieving the SDG's. This is partly due to a weak evidence base that does not give policy makers and programme managers the information needed to make decisions. Both international and national organisations should work together to fill the gaps in evidence and to gain a better understanding of what works and what does not in child well‐being.

#### 3.1.5 The intervention

The included interventions cover all main strategies to improve child well‐being outcomes. The seven intervention categories are:

1. Early child development
2. Health and nutrition
3. Education
4. Social work and welfare
5. Social protection
6. Environment health including Water, Sanitation and Hygiene (WASH)
7. Governance

Table 1 lists the intervention subcategories under each of these headings. The intervention domains of the mega‐map were informed by UNICEF's five key goal areas, linked to the UNICEF Strategic Plan (2018–2021).

- *Goal One: Every child survives and thrives*
- *Goal Two: Every child learns*
- *Goal Three: Every child is protected from violence and exploitation*
- *Goal Four: Every child lives in a safe and clean environment*
- *Goal Five: Every child has an equitable chance in life*

**Table 1:** Intervention categories and subcategories

Intervention category	Intervention subcategory
Early child development	Early childhood health intervention
	Early childhood nutritional interventions
	Early childhood education and parenting
	Women/maternal education and empowerment
Health and nutrition	Antenatal care, childbirth and postnatal care by Traditional Birth Attendants/Skilled Birth Attendants
	Childhood immunization
	Agricultural intervention/bio‐fortification
	Nutritional supplementation programme
	Management of severe acute malnutrition
	Community health interventions including community health workers
	Deworming
	Interventions for prevention and treatment of HIV/AIDS
	Prevention and management of childhood malaria
	Mass media campaigns on health education
	mHealth interventions for child health
	Maternal aid
	Mental health
Education	School voucher/reduced fees
	Decentralization and local community participation
	School feeding programme and mid‐day meal
	School based health interventions
	Systemic renewal
	Alternative schooling/nonformal education
	School sanitation and WASH
	Scholarship
	Teacher Incentiv es
	Teacher training
	Remedial education
	Pedagogical approach
Social work and welfare	Birth registration
	Child‐trafficking preventions
	Interventions for child abuse
	Gender based violence programme
	Substance abuse prevention
	Child protection services
Social protection	Social insurance schemes
	Labor market insurance
	Social assistance interventions
Environmental health including WASH	Improved sanitation and water
	Hygiene education
	Prevention of outdoor and indoor air pollution
	Prevention of environmental tobacco smoke
	Prevention of exposure to toxins such as lead, mercury and pesticides
	Safe places to play
	Traffic calming
**Governance**	Child rights
	Legislative reforms
	Child protection regulation

#### 3.1.6 Why it is important to develop the mega‐map

Evidence‐based research and multicountry experiences make a strong rationale for investing in child well‐being programmes. As evidence‐informed policymaking is seen to be of increasing importance, many agencies commission systematic reviews to inform policy, but due to lack of a central global repository, systematic reviews are often duplicated. Furthermore, they sometimes lack in quality to clearly and correctly inform policy and the existing evidence base around child well‐being has major gaps. Evidence maps are an approach to providing an overview of the available evidence, with various approaches adopted to evidence mapping by different agencies over the years (Saran & White, 2018). Since this map has such a broad scope—all of child well‐being—we label it as a mega‐map and map only systematic reviews and EGMs.

The mega‐map provides an overview of all available evidence syntheses on the key outcome domains and interventions aimed at improving child well‐being in LMICs using an intervention‐outcome framework. It helps to identify areas in which there are good bodies of synthesised knowledge to inform policy, and those areas in which there is little or no evidence synthesis. The map can help inform the identification of priority areas where evidence synthesis is currently lacking, such as rigorous studies of the effectiveness of early marriage interventions, child labour or the status of children in conflict‐affected situations. This will help create a central repository of all the available resources on the effectiveness of child well‐being.

UNICEF's Office of Research‐Innocenti, in collaboration with the Campbell Collaboration has published a set of five research briefs based around UNICEF's five strategic goal areas and highlighting the main findings from the mega‐map as well as remaining evidence gaps. As a subsequent step, the two organisations are worked together on an EGM on ending violence against children in LMICs.

#### 3.1.7 Existing EGMs and/or relevant systematic reviews

Since this is a mega‐map and has a very broad scope, it is the first map of its kind in this area. But there are related EGMs that are included in the mega‐map. Examples include:

1. EGM on social, behaviour and community engagement intervention produced by the WHO and the International Initiative of Impact Evaluation presents the evidence available on social, behavioural and community engagement interventions related to reproductive, maternal, newborn and child health programmes in LMICs (WHO, 2017).
2. EGM on primary and secondary education by 3ie that presents evidence on interventions designed to improve access to education and learning outcomes for primary and secondary school children in LMICs (International Initiative for Impact Evaluation [3ie]).
3. EGM on Intimate partner Violence highlights important gaps in the rigorous evidence base of intimate partner violence prevention programmes in LMICs (Ngeleka & UN, 2018).
4. EGM on adolescent well‐being by UNICEF that presents evidence related to protection, participation and financial and material well‐being of adolescents in LMICs (Bakrania, Ghimire, & Balvin, 2018).

There are a number of systematic reviews specifically focussed on child well‐being interventions such as:

1. Systematic review by (Bangpan, Chiumento, Dickson, & Felix, 2017) which assesses the impact of psychosocial interventions on the mental health of children and adults in humanitarian emergencies
2. Systematic review by (Miller, Donohue‐Dioh, Niu, Grise‐Owens, & Poklembova, 2019) which assesses home‐based child development interventions for preschool children from socially disadvantaged families
3. Systematic review by (Bright, Felix, Kuper, & Polack, 2017) which measures the effectiveness of interventions aimed at increasing access to health services for children aged 5 years and below in LMICs.

## 4 OBJECTIVES

### 4.1 Objectives

1. Develop a clear taxonomy of interventions and outcomes related to the interventions aimed at improving child well‐being outcomes in LMICs
2. Map available systematic reviews and EGMs on the interventions aimed at improving child well‐being outcomes in LMICs with an overview provided in a summary report
3. Provide database entries of included studies which summarise the intervention, context, study design and main findings.

## 5 METHODS

### 5.1 Defining EGMs

Campbell EGMs are based on a review of existing mapping standards (Saran & White, 2018) which drew in particular on the approach developed by 3ie (Snilstveit, Vojtkova, Bhavsar, & Gaarder, 2013). As defined in the Campbell Collaboration EGM Guidance, a mega‐map is a map of evidence synthesis, that is, systematic reviews, and does not include primary studies (Campbell Collaboration, 2020).

The primary dimensions are the rows and columns of the map which are, respectively, intervention categories (and subcategories) and outcome categories (and subcategories). Secondary dimensions, such as World Bank region, systematic review quality and population target groups were included as filters.

### 5.2 Types of evidence

The mega‐map includes only systematic reviews and EGMs of effects of interventions. The key characteristics for a review to be included as a “systematic review” are as follows:

1. A clearly stated set of objectives with predefined eligibility criteria for studies.
2. An explicit, search methodology.
3. A systematic search that attempts to identify all studies that would meet the eligibility criteria.
4. An assessment of the validity of the findings of the included studies, for example, through an assessment of risk of bias.
5. A systematic presentation, and synthesis, of the characteristics and findings of the included studies.

Relevant ongoing systematic reviews were also included but they were not assessed for quality. We did not include qualitative studies and the search was restricted to English language only.

We also included EGMs and it was defined as an approach for systematic presentation of all relevant evidence of a specified kind for a particular sector, subsector or geography. They typically contain systematic reviews and primary studies, but may include only one of these. There are many approaches to evidence mapping (Saran & White, 2018 and we included any map on child well‐being interventions.

### 5.3 Type of population

The primary population of interest for this mega‐map is children 18 years and under as per the definition by United Nation Convention (UNICEF, Convention on the Rights of the Child, 1989) and includes children from LMICs^1^).

Different child age ranges; infants (0–3 years), child (0–10 years), adolescent (10–18 years) were added as filters.

Population subgroups of interest include: children in conflict‐affected regions, children with disabilities, children from underrepresented communities (low income and ethnicity) and malnourished children.

### 5.4 Types of interventions/problem

The framework‐matrix of interventions and outcomes was primarily based on UNICEF's Strategic Plan (UNICEF, 2018).

The included interventions cover all main strategies to improve child well‐being outcomes. The seven main intervention categories are as mentioned below. The description for these categories is given in Annex 1.

1. Early child development
2. Health and nutrition
3. Education
4. Social work and welfare
5. Social protection
6. Environment health including WASH
7. Governance

### 5.5 Types of outcome measures

The categories cover seven broad outcomes as mentioned below:

1. Health
2. Healthy development
3. Learning and development
4. Risk factor reduction
5. Safety
6. Equity
7. Economic Impact

### 5.6 Search methods and sources

The mega‐map was developed in three stages:

- Stage 1: included a search of relevant systematic reviews and EGMs from 3ie databases. This initially included 99 studies.
- Stage 2: this mapped the studies from a full search of databases and included 302 systematic reviews
- Stage 3: (a) searched additional websites for grey literature, (b) consulted experts, and (c) screened additional submissions received in response to dissemination of the stage 2 map. This led to inclusion of 356 studies.

The search was as comprehensive as possible, using (but not limited to) relevant bibliographic databases and EGM databases, web‐based search engines, websites of specialist organisations and bibliographies of relevant reviews. Additionally, reference lists of the included reviews were reviewed.

#### 5.6.1 Databases

1. International Organisations
  – UNICEF
  – DFID (including Research for Development (R4D)
  – UNFPA Evaluation Database
  – WHO

2. EGM database
  – 3ie Evidence and Gap Map repository
  – Swedish Agency For Health Technology Assessment and Assessment of Social Services
  – Collaboration for Environmental Evidence
  – Global Evidence Mapping Initiative
  – Evidence based Synthesis Programme (Department of Veteran affairs)
  – Cochrane
  – Evidence based policing matrix
  – EPPI Centre Evaluation Database of Education Research

3. Systematic review database
  – Cochrane
  – Campbell Collaboration
  – 3ie Systematic Review Database
  – Research for Development

4. Academic databases
  – World Bank eLibrary (Ebsco)
  – The National Bureau of Economic Research (NBER)
  – Social Science Research Network (SSRN)
  – International Bibliography of Social Sciences (IBSS)
  – Applied Social Sciences Index and Abstracts (ASSIA)
  – Embase
  – PsycINFO
  – MEDLINE
  – ERIC

5. Grey Literature search/websites
  – World Health Organisation
  – World Bank
  – UNICEF
  – UNICEF Office of Research—Innocenti
  – UN Women
  – UNESCO
  – United Nations Population Fund
  – UN Economic and Social Council
  – CARE
  – Save the Children
  – African Development Bank
  – Young Lives
  – Association for the Development of Africa
  – Médians Sans Frontières
  – Action against Hunger
  – World for World Organization
  – Project Concern
  – One International
  – World Vision
  – Department for International Development
  – World Food Programme
  – Valid International
  – Concern Worldwide
  – Action Aid
  – Children's Investment Fund Foundation
  – International Red Cross
  – WHO ICTRP
  – Working Group on Early Childhood Development, Division for Social Policy & Development, Child Fund International
  – GreyNet International
  – Proquest Dissertations and Theses
  – Opengrey
  – Gates Foundation
  – Clinton Foundation
  – Abdul Latif Jameel Poverty Action Lab (J‐PAL)
  – Urban Youth Evidence Synthesis
  – Innovations for Poverty Action (IPA)
  – Child and Youth Finance International

A sample search is added as Annex 2.

### 5.7 Stakeholder engagement

The framework was developed through a consultative process. An advisory board was formed comprising of key experts in the area:

➢ Professor Anthony Costello, Director, Department of Maternal, Newborn, Child and Adolescent Health, World Health Organisation
➢ Dr. Ajay Khera, Deputy Commissioner In‐charge, Child and Adolescent Health, Ministry of Health and Family Welfare, India
➢ Dr. Julia St. Thomas, Deputy Director Strategic Initiatives, Violence Prevention and Response Unit, International Rescue Committee, UK.
➢ Dr. Laura Boone, Senior Technical Advisor for Child Protection International Rescue Committee, UK
➢ Mr. Laurence Chandy, Director of the Office of Global Insight and Policy, UNICEF
➢ Mr. Yehualashet Mekonen, Programme Manager, The African Child Observatory, African Child Policy Forum

In consultation with the expert group, intervention and outcome categories were agreed and used to set the scope of the mega‐map.

## 6 DIMENSIONS

### 6.1 Scope

Developing the scope is one of the critical stages in an EGM. Specifically, we identified intervention and outcomes to map that formed the EGM framework.

Stages in framework development:

1. The initial framework‐matrix of interventions and outcomes was developed based on UNICEF's strategic plan (UNICEF, 2018).
2. We then referred to strategic plan documents from other major funders and implementers on child well‐being programmes including UNESCO, WHO and the World Bank to identify any additional key intervention and outcomes of relevance.
3. The proposed framework was revised based on feedback received by advisory board (mentioned above).
4. We then pilot‐coded the framework using 30 key systematic reviews from the 3ie database and the framework revised further based on the exercise.
5. A mapping workshop was organised in London where key stakeholders and experts in the area were invited to participate. They reviewed the categories in an interactive exercise to fit the identified papers into the suggested categories and the feedback was reviewed.

The final framework aimed to provide an overview of existing systematic reviews and EGMs for child well‐being interventions such as:

1. Early childhood interventions that addressed the period from pregnancy, childbirth and children up to 3 years of age.
2. Health and nutrition interventions that addressed issues such as maternal health, timing and spacing of birth, childbirth, nutrition, prevention and treatment of childhood diseases.
3. Educational interventions that aimed to address improved learning and achievements and skills development of children from 4 to 18 years of age.
4. Social work and welfare interventions that aimed to protect the child from violence and other risk factors.
5. Social protection interventions that aimed to provide financial support to mother, children and families to access the basic amenities for survival and living.
6. Environmental WASH interventions to ensure that every child lives in a clean and safe environment.
7. Governance and advocacy intervention strategies to ensure sustainability.

#### 6.1.1 Conceptual framework

**Figure 1:**
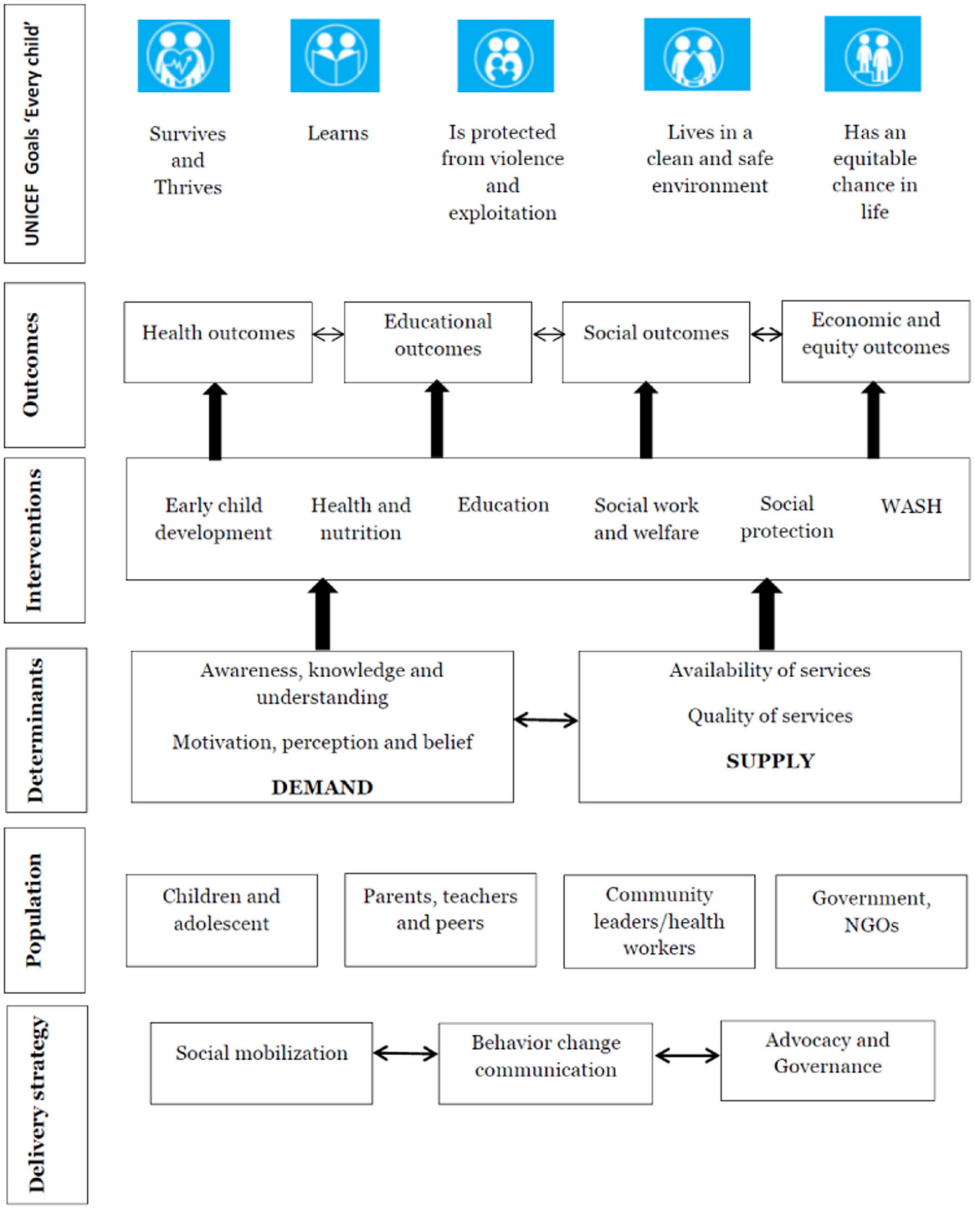
Conceptual framework of mega‐map. *Source*: Authors' own design (adapted from UNICEF's strategic goals)

Figure 1 presents a simplified theory of change which may underlie improvement in child well‐being outcomes. A crucial step is successful advocacy and social mobilisation. We assume that advocacy is one of the keys to setting priorities and improving policies to leverage child well‐being outcomes. Additionally, social mobilisation can help engage a broad range of actors so that they become involved in driving change, by raising awareness and by creating the demand for better policies, services and accountability. Both these components together help identify different target population and creating a demand and supply for better policies and services. Child well‐being encompasses numerous domains such as early child development, health and nutrition, education, social work and welfare, social protection and WASH and the interventions targeting each of them are represented in the conceptual framework. Following adoption of these interventions, several outcomes are then expected to improve such as on health, education, social, economic and equity. Since UNICEF's work is structured around five overarching areas of well‐being for every child who is grounded in the 2030 Agenda for Sustainable Development, improvement in any of these child well‐being outcomes, will directly or indirectly effect fulfilment of UNICEF's goals.

### 6.2 Description of intervention

The interventions included cover all main strategies to improve child well‐being outcomes.

Table 1 lists the intervention subcategories under each of these headings.

### 6.3 Description of population/geographic location

In addition to interventions and outcomes, the following filters were coded in the systematic reviews and mega‐map:

(1) Population subgroups of interest include: children with disabilities, children in conflict‐affected regions, children from underrepresented communities (low income, ethnicity, race) and malnourished children.
(2) Regions: East Asia and Pacific, Europe and Central Asia, Latin America and Caribbean, Middle East and North Africa, North America, South Asia, Sub‐Saharan Africa and conflict affected regions.
(3) Systematic review quality: Based on AMSTAR 2 as High, medium and low (Annex 3).
(4) Country classification by Income level: low‐income: <1,005; lower‐middle income: 1,006–3,955, upper‐middle income: 3,956–12,235 (World Bank, 2018).

## 7 DATA COLLECTION AND ANALYSIS

### 7.1 Screening and study selection

We imported the records from academic databases into our data management software EPPI‐Reviewer 4 (Thomas, Brunton, & Graziosi, 2010), and we used the built‐in tool to aid in removing duplicates. The grey literature was imported into and managed in Microsoft Excel due to reference format incompatibility with EPPI‐Reviewer 4.

The screening of studies in relation to inclusion/exclusion criteria was undertaken in two stages in EPPI‐reviewer.

The screening was carried out based on predefined eligibility criteria. We did not exclude any study based on study quality or outcomes (Table 2).

**Table 2:** Eligibility criteria for systematic reviews and EGMs

	Include	Exclude
Literature type	Systematic reviews and EGMs on interventions on child well‐being.	
	From:	– Reviews on clinical drug trials – Literature reviews – Rapid reviews – Prevalence reviews – Qualitative reviews – Association reviews – Commentary or editorials
	Published journal article	
	Grey literature:	
	– Working paper – Report – Thesis papers	
Population	– Studies on children 18 years and below. – Studies with multiple populations were included if they included children from age category as above. – Systematic reviews were included if the “inclusion criteria” in the review was to include the studies conducted in LMICs.	– Studies specifically on populations above 18 years – Systematic reviews for which studies from only high‐income countries were eligible for inclusion
Interventions	– Interventions to improve well‐being of children: health, education, social protection, social work and welfare, Governance	– No restriction
Publication date	– Studies published in and after year 2000	– Studies published before the year 2000

#### 7.1.1 Stage 1: Screen on title and abstract

Two independent reviewers screened studies and a third reviewer resolved the conflicts. In order to pass stage one, the title or abstract had to meet the eligibility criteria as listed in Table 2.

#### 7.1.2 Stage 2: Screen on full text

Full text documents were retrieved for all documents that passed stage one. Two reviewers independently evaluated all studies.

To ensure inter‐rater reliability, each reviewer was given two samples of 30 studies for classification. Results and discussion from one of the lead researchers were used as the standard for classification. An agreement rate of at least 70% was required. If the samples did not yield the required agreement, reviewers would discuss their responses to come to desired agreement rates.^2^

### 7.2 Data extraction and management

We used a standardised data extraction form (presented in Annex 4) to extract relevant information from reviews and EGMs. For each included study, two coders independently extracted the following information: bibliometric information, study descriptions, population and target groups, intervention, outcomes, regions and funders. All the included systematic reviews were subject to critical appraisal and quality rated.

### 7.3 Tools for assessing systematic review quality

We used a standardised checklist, AMSTAR‐2 (Shea, et al., 2017) to assess our confidence in the findings of each systematic review. The confidence ratings do not appraise the studies included in a review, but rather the methodology and reporting of the review. AMSTAR‐2 is a 16 item checklist and covers:

(1) Population, Intervention, Comparison, Outcomes and Study design (PICOS) in inclusion criteria, (2) ex ante protocol, (3) rationale for included study designs, (4) comprehensive literature search, (5) duplicate screening, (6) duplicate data extraction, (7) list of excluded studies with justification, (8) adequate description of included studies, (9) adequate risk of bias assessment, (10) reported sources of funding, (11) appropriate use of meta‐analysis, (12) risk of bias assessment for meta‐analysis, (13) allowance for risk of bias in discussing findings, (14) analysis of heterogeneity, (15) analysis of publication bias and (16) report conflicts of interest (Annex 3).

Items 2, 4, 7, 9, 11, 13 and 15 are termed “critical.” Study quality is rated high if there is no more than one noncritical weakness and medium if there is one or more noncritical weakness but no critical weakness. Studies with one or more critical weaknesses are rated low quality.

## 8 RESULTS

### 8.1 Description of studies

This section reports and discusses the 356 studies (333 systematic reviews and 23 EGMs) identified from our search and screening process for this EGM. The number of studies shown in each figure refers to the total number of studies falling under each category presented. Individual studies may be classified under multiple categories. For instance, if a study examines the impacts of multiple interventions, that study would add to the count for each intervention studied in that paper. The sum of studies for each figure may therefore be greater than the number of unique studies associated with that figure.

#### 8.1.1 Results of the search

**Figure 2:**
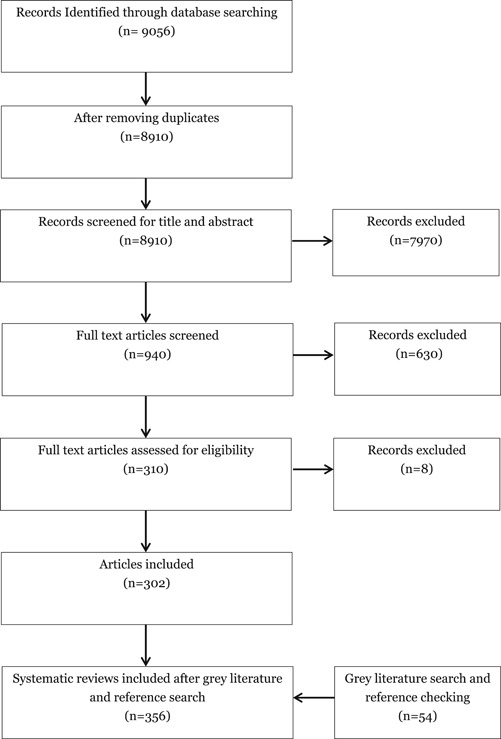
PRISMA for child well‐being mega‐map

As described in the PRISMA diagram (Figure 2), 9,056 records were initially identified as qualifying systematic reviews of evidence from LMICs from database searches. The screening process resulted in 302 systematic reviews and 16 EGMs to be included in Phase 1. In Phase 2, grey literature searches and reference/citation searching were performed which identified a further 76 studies to be included. After a further eligibility check, 22 of these were excluded.

As a result, a total of 356 records were included in the final map, of which 54 were identified from grey literature and reference checking. Of these, 333^3^ are systematic reviews and 23 are EGMs. All of the studies identified through the search and screening process were mapped/coded under the EGM framework. Hand searching of literature was not performed for this EGM.

### 8.2 Systematic review quality

**Figure 3:**
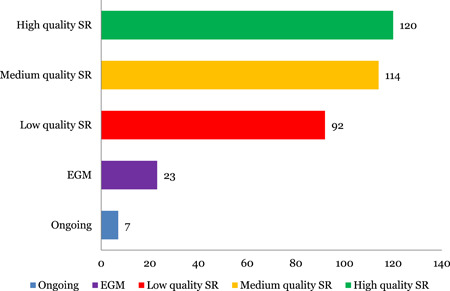
Number of systematic reviews and EGMs. EGM, evidence and gap map

Figure 3 shows the results of the critical appraisal of the included systematic reviews in the map and the number of EGMs and ongoing studies. Over half of the systematic reviews (50%) are rated as having high and medium confidence in study findings. The main reasons a study may be rated as low quality are (a) unclear inclusion and exclusion criteria (b) protocol was not registered before commencement of the review (c) the search strategy design was not sufficiently comprehensive (d) risk of bias was not considered when interpreting the results of the review (f) the search and screening procedures were not as per standard guidelines, and studies were insufficiently transparent with respect to methodology and did not provide the list and reasons for excluded studies.

### 8.3 Synthesis of included studies

#### 8.3.1 Publication of systematic reviews over time

**Figure 4:**
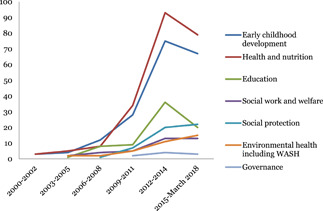
Trends in publication of systematic reviews by interventions over time

Figure 4 shows the number of completed systematic reviews covering child well‐being interventions published each year between 2000 and January to March 2018 (last search date for the map). The number of published systematic reviews peaked in the period 2012–2014 in all sectors of child well‐being interventions.

Health and nutrition was the most reviewed sector, followed by early childhood development.

#### 8.3.2 Publication of EGMs over time

**Figure 5:**
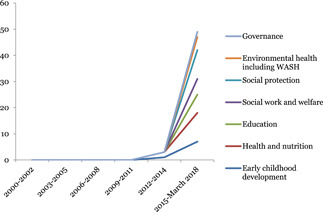
Trends in publication of EGMs by interventions over time. EGM, evidence and gap map

Figure 5 shows the number of completed EGMs covering child well‐being interventions published each year between 2000 and March 2018. The number of EGMs published showed a striking peak in 2012–2014. This is possibly because 3ie started commissioning EGMs from 2010 onwards and many organisations like IRC adopted the 3ie methodology after 2010. Education was the most reviewed sector followed by health and nutrition and social protection.

#### 8.3.3 Distribution of studies across Intervention and outcome categories

The health quadrant of the EGM which maps the studies of the effects of health interventions on health outcomes—is the most heavily populated section of the map (Tables 3 and 4).

**Table 3:** Aggregate map: Number of systematic reviews by intervention and outcome categories

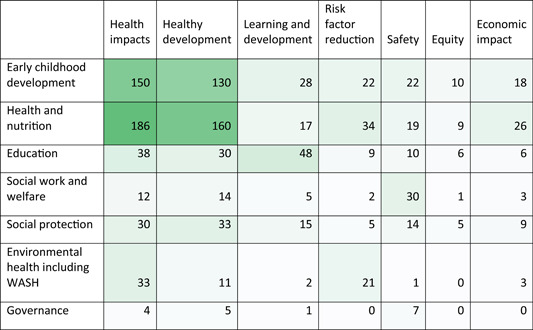

^a^ Colour saturation shows concentration of evidence from systematic reviews.

**Table 4:** Aggregate map: Number of EGMs by intervention and outcome^a^ categories

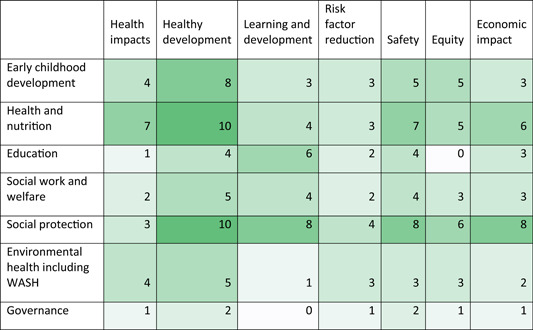

^a^ Colour saturation shows concentration of evidence from EGMs.

#### 8.3.4 Distribution of studies across early childhood development

##### Systematic reviews

**Figure 6:**
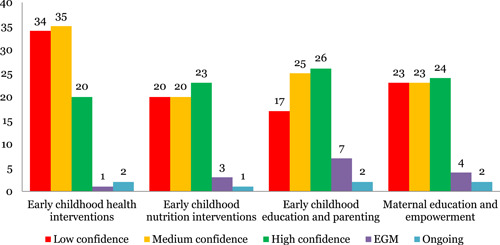
Number of studies for early childhood development interventions by study theme and quality

This is a fairly concentrated area of the map with a good amount of evidence in all intervention sub‐categories; early childhood health intervention (93), maternal education and empowerment (78), early childhood education and parenting (69) and early childhood nutritional interventions (65). The main outcomes noted were health impacts (152) and healthy development (132). Limited evidence was identified on learning and development (28), risk factor reduction (220 and economic impact (18) of these interventions. Only one systematic review was identified with an equity focus. About 30% of the reviews were identified to be of low quality (Figure 6).

##### EGMs

There were seven EGMs identified covering early childhood education and parenting, four on maternal education and parenting, three on early childhood nutritional interventions and one on early childhood health interventions.

Healthy development (eight) was the most commonly studies outcome, five on safety and five assessed equity as an outcome measure. Four EGMs assessed impact on health and three on learning and development and risk factor reduction each.

#### 8.3.5 Distribution of studies across health and nutrition

##### Systematic reviews

This is the most populated area of the map, but studies were unevenly distributed across intervention subcategories. Systematic reviews were concentrated in; community health interventions including Community Health Workers (CHWs; 138) and Antenatal care and postnatal care by Traditional Birth Attendants (TBA)/Skilled Birth Attendant (SBA) (108) and nutritional supplementation programme (62).

The least populated areas were management of severe acute malnutrition (8), deworming (9), agricultural interventions/bio‐fortification (13), m‐Health interventions for child health (16), prevention and management of childhood malaria (17) and mental health programmes (23).

The outcomes covered by systematic reviews mainly fall in two domains, health impact (188) and healthy development (162). About 26 studies were identified assessing the economic impact of these interventions. Only nine studies were identified assessing equity outcomes. Over 100 studies relate to the main outcomes of mortality, morbidity, disability and childhood illness and nutrition. There were fewer studies relating to risk factors notably on childhood injuries, alcohol and substance abuse, handwashing, clean environment, diet and physical activity.

**Figure 7:**
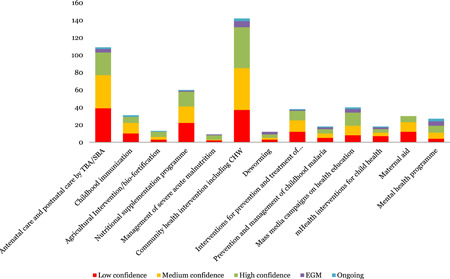
Number of studies for health and nutrition interventions by study theme and quality

In terms of quality, 26% of child health reviews are classified as low‐quality (Figure 7).

##### Evidence and gap maps

Thirteen EGMs were identified in the area of health and nutrition, with the majority covering community health interventions including CHWs (7), mental health programmes (5), antenatal care and postnatal care by TBA/SBA (4), mass media campaigns on health education (4). Healthy development (10) is the most common outcome covered, followed by health (7) and economic impacts (6) and five (5) assessing equity.

#### 8.3.6 Distribution of studies across education

##### Systematic reviews

The most studied interventions in the education domain were school based‐health interventions (38), pedagogical approaches (36), alternative schooling/nonformal education (27), teacher training (27), decentralization and local community participation (26), teacher incentives (19) and remedial education (16). Limited systematic reviews were identified on school voucher/reduced fees (10) and systemic renewal (6).

Learning and development is the most studied outcome (48), followed by health impacts (38) and health development (30). Only six systematic reviews studied the economic impacts of educational interventions. For learning and development outcomes, the largest number of studies relates to cognitive development (50) and learning and achievement (58). Only six reviews had an equity focus.

**Figure 8:**
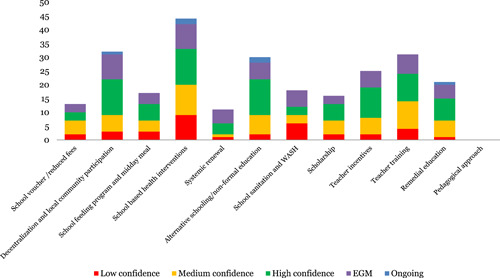
Number of studies for education interventions by study theme and quality

In terms of quality, 22% of educational reviews were identified to be of low quality (Figure 8).

##### Evidence and gap maps

School based interventions (nine) and decentralization and local community participation (nine) and teacher training (seven) were the most covered interventions by EGM. Alternative schooling, school sanitation and WASH and teacher incentives and pedagogical approaches were covered by six EGMs each. No EGMs were identified assessing equity component.

#### 8.3.7 Distribution of studies across social work and welfare

##### Systematic reviews

This is a scarcely populated domain in the map. The largest concentration of reviews in this area were on gender‐based violence interventions (21), also termed as violence against children and women, followed closely by interventions on child abuse (13) and child protection services (13). There was a lack of evidence on child trafficking interventions (one) and substance abuse prevention (two). The outcomes covered by systematic review were concentrated in three outcome domains—safety (30), health (13) and healthy development (15). Only three studies measured the economic impact of these interventions. On safety outcomes, violence against children (35) was the most studied outcome. There was limited evidence on child rights (eight) and FGM prevalence (four). Insufficient evidence was found on child labor and child marriage. There considerable lack of reviews in this intervention areas with explicit focus on equity (one).

**Figure 9:**
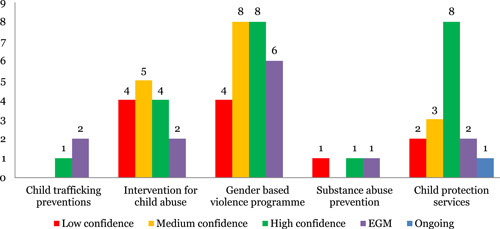
Number of studies for social work and welfare by study theme and quality

Twenty‐two percent of reviews were identified to be of low quality (Figure 9).

##### Evidence and gap maps

Gender‐based violence interventions (six) were the most covered by EGMs, followed by interventions on child abuse (two), child trafficking interventions (two) and child protection services (two). Safety (five) is the most studied outcome in EGMs as well, followed by health (four) and healthy development (five) and equity (three).

#### 8.3.8 Distribution of studies across social protection

##### Systematic reviews

Social assistance intervention (44) is the most concentrated intervention area, followed by social insurance schemes (13). There was lack of evidence on labour market insurance (one).

Health (31) and healthy development (34) were the most studied outcomes, followed by learning and development (15) and safety (14). Economic impact was assessed in nine reviews and no reviews were identified on equity component.

**Figure 10:**
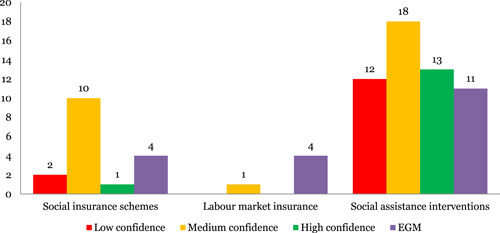
Number of studies for social protection by study theme and quality

24% of the reviews were identified to be of low quality in this area (Figure 10).

##### Evidence and gap maps

Social assistance (11) was the most covered intervention area followed by social insurance schemes (four) and labour market insurance (four).

Healthy development (10), learning and development (eight) and safety (eight) and equity (six) were the most covered outcomes.

#### 8.3.9 Distribution of studies across environmental health including WASH

##### Systematic reviews

On the interventions, 24 reviews were identified on improved sanitation and water and 21 on hygiene education. There was limited evidence on prevention of environmental tobacco smoke (seven), safe places to play (three), prevention of outdoor and indoor air pollution (one) and traffic calming (one).

On the outcomes, health impacts (33) and risk factor reduction (21) was the most commonly studies outcomes, followed by health development (11). Limited evidence was found assessing impact on equity (six), learning and development (two), safety (one), and economic impact (three) and only one review assessing equity.

**Figure 11:**
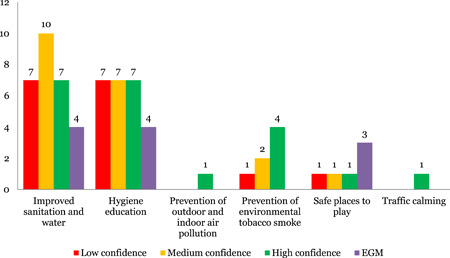
Number of studies for environmental health including WASH by study theme and quality

Nine reviews were assessed to be of low quality and 13 medium of quality (Figure 11).

##### Evidence and gap maps

Four EGM were identified each on improved sanitation and water and hygiene education and three on safe places to play.

On the outcomes, health (four) and healthy development (four) was the most commonly studied outcomes, followed by risk factor reduction (three), safety (three), equity (three) and economic impact (two).

#### 8.3.10 Governance

##### Systematic reviews

Governance was the least studied area of the maps with only six reviews on legislations on child rights (six) and four on child protection legislations (four). Safety (seven) was the most studies outcome, followed by health development (five) and impact on health (four). No studies were identified on equity or economic impact.

##### Evidence and gap maps

**Figure 12:**
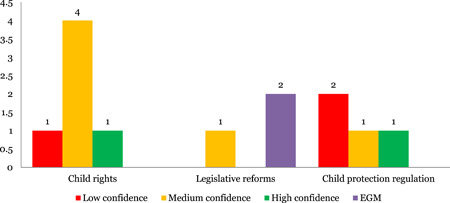
Number of studies for governance by study theme and quality

We identified only two EGMs on child protection legislations. These EGMs assessed impact on safety (2), healthy development (2), and one each on health, risk factor reduction and economic impact. One EGM assessed equity outcomes (Figure 12).

#### 8.3.11 Studies by UNICEF strategic goals

**Figure 13:**
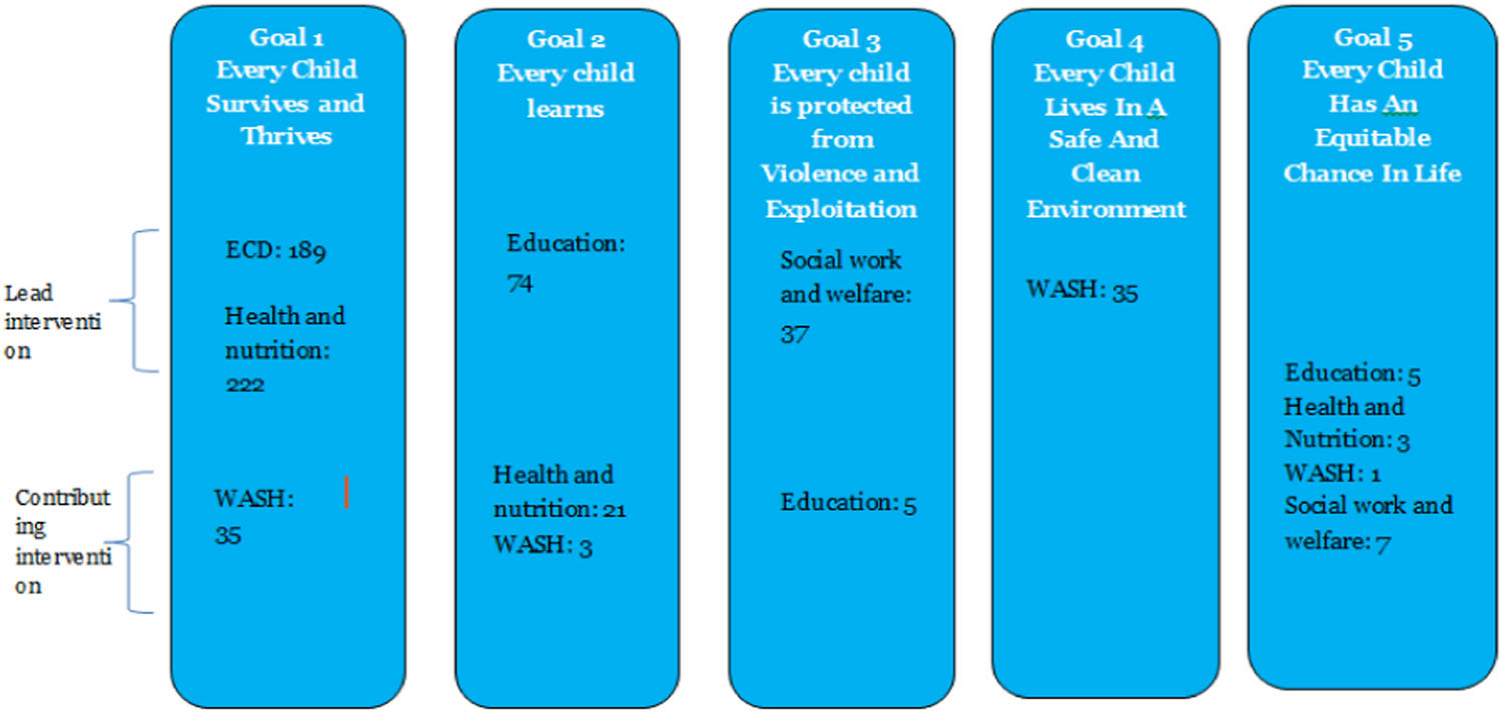
Number of studies by UNICEF strategic goals

Figure 13 shows areas in which there is ample evidence for UNICEF strategic goals related to child well‐being. As clearly stated above, Goal 1 (Every child survives and thrives) and Goal 2 (Every child learns) are heavily populated. Goal 3 (every child is protected from violence and exploitation) and Goal 4 (every child lives in a clean and safe environment) were less populated and there is scant evidence in Goal area 5 (every child has an equitable chance in life).

### 8.4 Additional dimensions

#### 8.4.1 Equity

There are thirteen systematic reviews in the mega‐map with an explicit equity focus. That is, reviews which either address equity or focus on a disadvantaged group, such as people with disabilities. These are concentrated in health and nutrition (six), and education intervention (five) areas. There is just one study with respect to early child development, and none at all for social protection or rights and governance. The 13 reviews identified as addressing equity report a broad range of outcomes. Most of these outcomes relate to health and education. However, there are also a number related to the UNICEF goal that every child is protected from violence and exploitation, with child abuse and neglect being reported in three of the 13 studies.

Approximately half (six out of 13) of the studies are rated as high quality.

#### 8.4.2 Population subgroups

**Figure 14:**
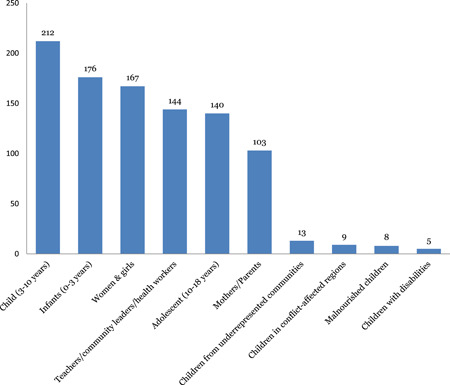
Number of studies by population subgroup

There is a fair amount of evidence assessing the effectiveness of interventions for all age‐groups, though it is highly concentrated for children in the young child (249). There was limited evidence focusing specifically on vulnerable populations such as children with disabilities (12), children in conflict‐affected regions (11) and malnourished children (8). There were limited systematic reviews focusing on gender and equity issues (Figure 14).

#### 8.4.3 Region

**Figure 15:**
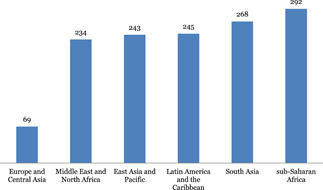
Number of systematic reviews by region

Systematic reviews were found to be almost equally concentrated in all regions with sub‐Saharan Africa (292) and South Asia (268) having the highest number of reviews. These were closely followed by Latin America and Caribbean (245), East Asia and Pacific (243) and Middle East and North Africa (234) (Figure 15).

#### 8.4.4 Funders for systematic reviews

**Figure 16:**
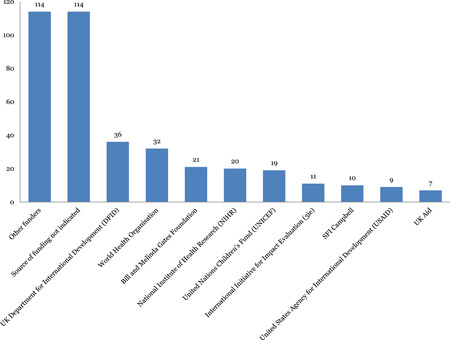
Funders of systematic reviews

Figure 16 provides a breakdown of funders of the systematic reviews included in the mega‐map. Sixty‐five percent (216) of studies provide information about the systematic review funder. The United Kingdom's DFID is the most cited funder (36) followed by the WHO (32), Bill and Melinda Gates foundation (21), NIHR (20) and UNICEF (21). The “other funders” category^4^ in the figure below includes funders such as AusAid/DFAT (six), Hewlett Foundation (six), SFI Campbell (six), Wellcome Trust (six), MacArthur Foundation (five), Australian Cochrane centre (five).

**Figure 17:**
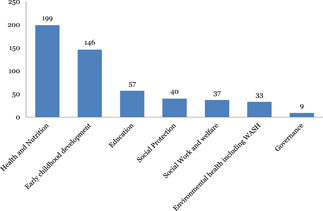
Most funded systematic review sectors

Figure 17 shows that the most funded systematic review areas have been health and nutrition and early childhood development. International organisations, national governments, research institutes, and donors should consider funding more systematic reviews in areas such as social protection, social work and welfare and environmental health/WASH in order to address key gaps in knowledge and enhance the child well‐being agenda and the achievement of the SDGs.

#### 8.4.5 Funders for EGMs

**Figure 18:**
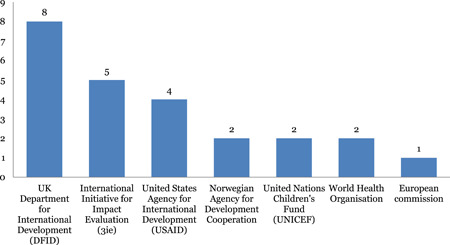
Funders of evidence and gap maps

Figure 18 provides a breakdown of funders of the included EGMs. The United Kingdom's DFID is the most cited funder (eight) followed by USAID (four). The most cited producer is the 3ie.

## 9 DISCUSSION AND GAPS IN EVIDENCE

### 9.1 Summary of main results

The main finding reported from the mega‐map is that there is good amount of evidence in more traditional areas of study such as health and nutrition, education and early childhood development. However, there is an uneven distribution of evidence across intervention and outcome subcategories. Though health and nutrition were the most populated areas of the map, there were gaps identified in many subintervention areas; management of severe acute malnutrition, agricultural intervention/biofortification, m‐health intervention for child health. In education, there is limited evidence around remedial education, nonformal education and scholarships and systemic intervention. In terms of quality, about 26% of the systematic reviews were identified to be of low quality for health and nutrition. This was found to be similar for early childhood development (30%) and education (22%). In terms of outcomes, health and learning were found to be the most studied as well; limited studies were identified assessing the impact of interventions on risk reduction, safety, equity and economic outcomes.

Striking gaps in evidence were identified in nontraditional areas such as social protection and social welfare and Governance with a lack of evidence in many areas such as in prevention of child trafficking, substance abuse, child abuse and in social insurance and labour market insurance. These areas were not just under‐researched but also under funded. The most funded systematic review areas have been health and nutrition and early childhood development over the years. The major funders have been the DFID, WHO, the Bill and Melinda Gates Foundation, NIHR and UNICEF.

### 9.2 Areas of major gaps in the evidence

#### 9.2.1 Overall gaps

**Figure 19:**
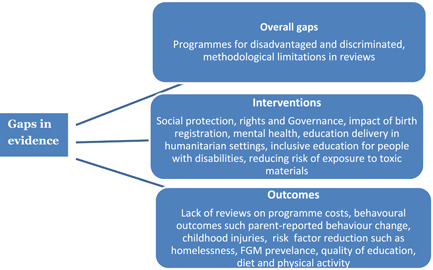
Priority areas and gaps in evidence

Some priority areas and gaps in evidence are listed in (Figure 19). There priorities emerge from both the gaps in the map, and stakeholder consultations on the map findings.

– There is a clear gap in reviews which are explicitly focused either on equity or on programmes for disadvantaged groups and those who are discriminated against such as children with disabilities, children belonging to ethnic minorities and low income groups.
– Very few reviews were identified that conducted cost‐analysis.
– The evidence synthesis which is available is mostly in the areas of health and education. More is needed in those areas. But more still is needed in other areas where there are limited reviews such as governance.

#### 9.2.2 Gaps by each sector

##### Health

One major gap is for mental health interventions and mental health outcomes in LMIC's. Mental health has been recognised as a major development challenge for some time (WHO, 2010), but the evidence is lagging behind. There are studies for early child interventions, which mostly measure effects on psychosocial development rather than mental health problems. Furthermore, an EGM of disability in LMICs (Saran, White, & Kuper, 2020) showed primary studies to be concentrated in very few countries, and few studies addressed the social aspects of disability necessary for inclusive development.

A gap was also identified on management of severe acute malnutrition, which is a huge issue in many LMICs. Since undertaking this map, the Bill and Melinda Gates Foundation has commissioned ten Campbell systematic reviews in this area.

There is less information on behavioural outcomes—that is parent‐reported behaviour change of children and the impact of diet and physical activity on child health. Parenting practices are a growing area of interest in developing countries. The International Rescue Committee (IRC), has promoted them based on evidence from developed countries. Impact evaluations of IRC's projects show good results, but reviews are needed to stay abreast of the growing evidence base.

##### Education

Though, there are no absolute gaps in evidence in this domain, there was limited evidence on systemic renewal and on school vouchers/reduced fees. There is no systematic review of delivering education in humanitarian settings and of inclusive education for people with disabilities. More reviews are needed on the impact of education system strengthening. The 3ie education EGM (which is included in the mega‐map) gives a more detailed picture showing low numbers of primary studies for scholarships and user fees, information interventions and remedial education, as well as a lack of studies for many teacher and cognitive outcomes.

##### Social work and welfare

Social work and welfare in LMICs is an under‐researched area in child well‐being. There is a lack of studies on child trafficking, early marriage, FGM and child labour. There was also limited evidence relating to child abuse and neglect as well as impact of birth registration. As a follow up to this map, UNICEF has commissioned Campbell Collaboration to undertake a map of Violence Against Children (Pundir, Saran, White, Adona, & Subrahmanian, 2019).

There were no studies identified highlighting the impact of birth registration on child safety and well‐being.

##### Social protection

Social protection is well covered because of the amount of reviews on conditional cash transfers, but there is a lack of evidence on social insurance and labour market insurance. Micro insurance programmes offer a financially sustainable model of social protection so more understanding of their effectiveness is important to inform policy.

##### Risk factors and waSH

Child injuries are an important area for which there are few reviews. There are also no reviews of safe places to play and traffic calming despite the fact that over 90% of traffic fatalities are in developing countries. There is a new ongoing map on road safety, from which preliminary findings show over 90% of evidence comes from North America. So primary studies of traffic safety measures in LMICs are an important gap.

There are no or few studies to help identify effective strategies to reduce the exposure of children to indoor and outdoor air pollution, to reduce the risk of accidents including road‐related accidents, or to reduce risk of exposure to toxic materials.

### 9.3 Limitations of the EGM

The search strategy was systematic but owing to the large scope of the map, there are limitations. Some studies may have been missed, although several steps were taken to reduce this risk. For example, the search was conducted across a wide range of academic databases and the search was validated by an expert; reference and citation checking was undertaken and key donor websites were searched to identify grey literature. However, hand‐searching of literature was not undertaken.

Although we used a consultative process that included two roundtable events to develop the framework, experts did not fully agree on all the categories. For example, there were comments that child protection services and interventions for child abuse should be a single category. Similarly, child right interventions were not fully agreed on. We worked based on the definitions we had, which could have led to judgement bias for some studies.

## 10 AUTHORS' CONCLUSIONS

### 10.1 Implications for research

The mega‐map demonstrates major gaps in our understanding, suggesting many practices in the promotion of child well‐being are largely unsupported by evidence.

The mega‐map is the first step in identifying priority areas for rigorous systematic reviews and EGMs on child well‐being interventions. Based on the findings, a systematic research prioritisation should now be undertaken. As an immediate development from the mega‐map, UNICEF‐Innocenti and Campbell Collaboration are now working on an EGM on ending violence against children in low and middle income countries (Pundir et al., 2019). A similar collaboration is now needed in all the areas of weak evidence synthesis to help improve and advance research.

➢ There were gaps in reviews with explicit focus on equity however, there may be review of reviews that contain relevant evidence, but mapping that requires diving into the review contents in more detail. A next step could be to produce an equity‐focused version of the mega‐map which allows identification of which reviews present evidence disaggregated for priority groups.
➢ It would be useful for global and country partners to work together to achieve consensus on priority areas for evidence synthesis and to adopt a coordinated approach to undertaking and updating maps and reviews across priority areas.
➢ Efforts are also needed to identify optimal study design, key interventions and outcome areas so that a universal evidence base can be built and used.
➢ There were a few dated systematic reviews that were identified and these need updating. Examples include in the areas of FGM, early marriage and child labour.
➢ There is a good amount of high‐quality systematic reviews in health and education and this implies that there is a substantial evidence base for governments and international agencies to draw upon for programme and policy design. The map is a starting point in guiding people to the evidence: further analysis and knowledge brokering is required for the evidence to be usable.
➢ More evidence synthesis are needed to fill an important gap in measuring interventions to meet the needs of vulnerable populations with important considerations including equity, gender, ethnicity, humanitarian settings and income groups.

## ROLES AND RESPONSIBILITIES

S. A., A. K., and W. H. contributed to writing and revising this report. The search strategy was developed and piloted by S. A. and validated by E. J. Search was conducted by S. A. S. A will be responsible for updating this EGM.

## CONTRIBUTIONS OF AUTHORS

- **Content expertise**

K. A. has experience regarding generation, communication and use of evidence across all of UNICEF's research and policy areas. H. W. has published papers, including systematic reviews and impact evaluations, on various aspects of child well‐being.

- **EGM methods expertise**

A. S. and H. W. have previous experience in systematic review methodology, including search, data collection, theory‐based synthesis. They carried out the various processes required in an EGM, such as search, eligibility screening, quality assessment and coding. J. A. is an experienced screener and helped in screening and coding of included studies.

- **Information retrieval expertise**

A. S. has training in designing and implementing search strategies and she performed the searches for the mega‐map. The search strategy was validated by John Eyers, an expert with over 15 years' experience

## DECLARATIONS OF INTEREST

None.

## PLANS FOR UPDATING THE EGM

With thanks to financial support from the Bill and Melinda Gates Foundation, the mega‐map and associated research briefs will be updated annually over the lifetime of the UNICEF Strategic Plan 2018–2021, thus making the resource an open‐access, living map.

## SOURCES OF SUPPORT

The mega‐map is funded by UNICEF's Office of Research‐Innocenti.

## Supporting information

Supporting information

## Footnotes

^1^
LMICs are defined by World Bank as low‐income economies—those with a Gross National Income (GNI) less than $995; lower middle‐income economies—those with a GNI per capita between $996 and $3,895; and upper middle‐income economies—those with a GNI per capita between $3,896 and $12,055 (2018).

^2^
Ideally the agreement rate should be 80% between reviewers. However, for this map we had to work with 70% agreement rate for coding as even with repeated rounds of discussion and number of revisions in categories, the team was unable to reach 80% agreement, mainly due to overlap in health related intervention and outcome categories.

^3^
Note the large number of studies identified in the search is due to the broad scope of the map and hence the search had to be comprehensive.

^4^
All Other funders not listed above funded less than seven maps of reviews.

## ANNEX 1 DESCRIPTION OF INTERVENTION CATEGORIES AND SUBCATEGORIES

**Table:**

**Early childhood development**	
*Encompasses physical, socioemotional, cognitive and linguistic development between 0 and 8 years of age*	
Early childhood health intervention	This includes initiatives in health care, including health service provision, disease prevention, and health promotion to provide the continuum of maternal and child pre‐ and postnatal care
Early childhood nutritional interventions	This includes initiatives to ensure that pregnant women, breastfeeding mothers, and young children are adequately nourished
Early childhood education and Parenting	This includes interventions that provide opportunities for children to interact with responsive adults and actively learn with peers to prepare for primary school entry and also interventions directed on parent training
Women/Maternal Education and empowerment	Interventions working with the mothers and families to change parents' or caregivers' knowledge, attitudes and behaviours or to encourage dialogue on health care services and decision making by women
**Health and nutrition**	
*A health intervention is an act performed for, with or on behalf of a person or population whose purpose is to assess, improve, maintain, promote or modify health, functioning or health conditions*	
Antenatal care, childbirth and post‐natal care by TBA/SBA	Antenatal care is the care and help received from health professionals/birth attendants during the course of pregnancy. Postnatal care extends for between 6 and 8 weeks after the birth of child. During this period, routine checks are carried out by the health visitor and recovery is monitored
Childhood immunization	Immunization is the process whereby a person is made immune or resistant to an infectious disease, typically by the administration of a vaccine
Agricultural intervention/biofortification	Biofortification is the process by which the nutrient density of food crops is increased through conventional plant breeding, and/or improved agronomic practices and/or modern biotechnology without sacrificing any characteristic that is preferred by consumers or most importantly to farmers. It is recognized as a nutrition‐sensitive‐agriculture intervention that can reduce vitamin and mineral deficiency
Nutritional supplementation programme	Interventions to promote supplementation of nutrients such as iron folic supplementation in pregnant women
Management of severe acute malnutrition	Interventions in children who are 6–59 months of age, severe acute malnutrition is defined by a very low weight‐for‐height/weight‐for‐length, or clinical signs of bilateral pitting oedema, or a very low mid‐upper arm circumference
Community health interventions including CHWs	Community health workers (CHW) are members of a community who are chosen by community members or organizations to provide basic health and medical care to their community capable of providing preventive, promotional and rehabilitation care to these communities. Other names for this type of health care provider include village health workers, community health aides, community health promoters, and lay health advisors
Deworming	Periodic treatment with anthelminthic (deworming) medicines, without previous individual diagnosis to preschool‐ and school‐aged children living in endemic areas
Interventions for prevention and treatment of HIV/AIDs	Interventions related to HIV and other STIs among adolescents, including testing, incidence and prevalence
Prevention and management of childhood malaria	Providing health and/or counselling services specific to childhood malaria in a community setting
Mass media campaigns on health education	Interventions employing mass media (e.g., radio and television) to deliver health focused messages
Maternal aid	Interventions that aim to provide subsidies that affect maternal and child health outcomes
**Education**	
*Support needed to acquire the skills being taught by the educational system and should address functional skills, academic, cognitive, behavioural and social skills that directly affect the child's ability to access education*	
School voucher/reduced fees	Programmes providing allowances to cover all or some of the costs associated with education, including school fees, uniforms and books
Decentralization and local community participation	Interventions improving the participation of the community in effectively promoting education
School feeding programme and mid‐day meal	The programmes supplies free lunches on working days for children in primary and upper primary classes in government, government aided local body, Education Guarantee Scheme, and alternate innovative education centres
School‐based health interventions	School health and nutrition programmes that help children to complete their education and develop health knowledge and lifelong positive behaviours. These interventions address nutrition, hygiene, oral health and communicable diseases
Systemic renewal	Systemic Renewal is “about continuous, critical inquiry into current practices, identifying innovations that might improve education, removing organizational barriers to that improvement, and providing a system structure that supports change”
Alternative schooling/nonformal education	Nonformal education refers to education that occurs outside the formal school system. Nonformal education is often used interchangeably with terms such as community education, adult education, lifelong education and second‐chance education. It refers to a wide range of educational initiatives in the community, ranging from home‐based learning to government schemes and community initiatives. It includes accredited courses run by well‐established institutions as well as locally based operations with little funding
School sanitation and WASH	Interventions to ensure child friendly water supply, toilet and hand washing facilities in the schools and promote behavioural change by hygiene education
Teacher Incentives	Seek to improve the working conditions in schools so that teachers are motivated to come to work and improve their performance
Remedial education	Also known as developmental education, basic skills education, compensatory education, preparatory education, and academic upgrading, is assigned to assist students in order to achieve expected competencies in core academic skills such as literacy and numeracy
Pedagogical approach	Interactions between teachers, students, and the learning environment and the learning tasks. This broad term includes how teachers and students relate together as well as the instructional approaches implemented in the classroom. Types include teacher‐centred, learner‐centred and learning centred pedagogy
**Social work and welfare**	
*Development and provision of public or private social services to promote social justice amongst individuals and groups of individuals. While the term social welfare refers more generally to the well‐being of groups and individuals as well as the system of social service delivery, the term social work refers more specifically to the professional practice of delivering these social services*	
Child protection services	Child Protective Services (CPS) is a branch of a state's social services department that is responsible for the assessment, investigation and intervention regarding cases of child abuse and neglect, including sexual abuse. CPS typically takes cases where a child has been abused or is believed to be at risk of abuse by someone who has caregiving responsibilities for that child
Intervention for child abuse	Includes clinical treatment of physical and psychological injuries, family counselling, self‐help services, the provision of goods and services such as homemaker or respite care, legal action against the perpetrator, and removal of the child or the offender from the home
Child‐trafficking prevention/interventions	Supporting training of professionals working with children including social workers, health workers, police and border officials to effectively deal with trafficking. Providing children with social services, health care, psychosocial support, and reintegration with family and community
Substance abuse prevention	Programmes and strategies for substance abuse preventions among children and adolescents
Birth registration	
**Social protection**	
*Is defined as the set of policies and programmes designed to reduce poverty and vulnerability by promoting efficient labour markets, diminishing people's exposure to risks, and enhancing their capacity to protect themselves against hazards and interruption/loss of income*	
Social insurance schemes	Microfinance, employability training, vocational training and savings programmes that aim to affect child well‐being outcomes
Labour market interventions	Interventions covering work‐related injuries of employees, broadly affecting household income generation and child health
Social assistance interventions	Unconditional or conditional cash transfer programmes that aim to affect child well‐being outcomes
**Environmental health including WASH**	
Improved sanitation and water	Refers to access to adequate and equitable sanitation and hygiene for all and end open defecation, paying special attention to the needs of women and girls and those in vulnerable situations
Hygiene education	Setting up a comprehensive educational programme to ensure that the community:
	– is aware of the importance of water quality and its relation to health, and of the need for safe water supplies;
	– accepts the importance of surveillance and the need for a community response;
	– understands and is prepared to play its role in the surveillance process;
	– has the necessary skills to perform that role.
Prevention of outdoor and indoor air pollution	There are myriad forms and sources of indoor pollution, including combustion (tobacco smoke, stoves, fireplaces, and wood stoves), household products, construction materials, biologic agents (e.g., microbes, pets), off‐gassing from water, and soil gas. In particular, soil gas is the origin of most indoor radon
Prevention of environmental tobacco smoke	Includes intervention such as to reduce exposure to ETS (smoking bans and restrictions), interventions to reduce tobacco‐use initiation (increasing the unit price for tobacco products and multicomponent mass media campaigns), and interventions to increase cessation (increasing the unit price for tobacco products; multicomponent mass media campaigns; provider reminder systems; a combined provider reminder plus provider education with or without patient education program; multicomponent interventions including telephone support for persons who want to stop using tobacco; and reducing patient out‐of‐pocket costs for effective cessation therapies)
Prevention of exposure to toxins such as lead, mercury and pesticides	Includes all the strategies to protect children from existing toxins, such as lead, mercury, and tobacco such as the US EPA and FDA need more authority and resources to regulate and reduce emissions and exposures
Safe places to play	Creating safe places for children like playgrounds, where they are protected from violence, road accidents and other injuries
Traffic calming	Includes strategies as speed reducing initiatives near urban residential areas include speed cushions, humped pelican crossings, raised junctions, narrowing the road(s), gateways at the entrances to the area, build‐outs to protect on‐street parking spaces, and mini‐roundabouts
**Governance**	
*Governance can be seen as the exercise of economic, political and administrative authority to manage a country's affairs at all levels. It comprises the mechanisms, processes and institutions, through which citizens and groups articulate their interests, exercise their legal rights, meet their obligations and mediate their differences*	
Child rights	It provides a set of principles and standards covering
	Children's entitlements to such essentials as education, healthcare and the right to be heard, as well as protection from abuses such as unjust treatment and exploitation
	It places an obligation on states to ensure that all children within their jurisdiction (including noncitizens such as refugees) enjoy these rights
Legislative reforms	Develop and adopt national legislation to implement child protection laws
Child protection regulation	Measures that protect access to resources, promote employment, and support the childcare role

## ANNEX 2 SEARCH STRATEGY

**Table:**

Search string/Key words (OVID platform)	
Developing Country Free Text	
– (developing OR less‐developed OR less* developed OR "under developed" OR underdeveloped OR under‐developed OR middle‐income OR "middle income" OR "low income" OR low‐income OR underserved OR "under served" OR deprived or poor*) adj3 (countr* OR nation OR population OR world OR state OR economy OR economies).mp – ("third world" OR L&MIC OR L&MIC OR LAMIC OR LDC OR LIC OR lami countr* OR transitional countr*).mp – (Africa OR "Sub‐Saharan Africa" OR "North Africa" OR "West Africa" OR "East Africa" OR Algeria OR Angola OR Benin OR Botswana OR Burkina Faso OR Burundi OR Cameroon OR "Cape Verde" OR "Central African Republic" OR Chad OR "Democratic Republic of the Congo" OR "Republic of the Congo" OR Congo OR "Cote d'Ivoire" OR "Ivory Coast" OR Djibouti OR Egypt OR "Equatorial Guinea" OR Eritrea OR Ethiopia OR Gabon OR Gambia OR Ghana OR Guinea OR Guinea‐Bissau OR Kenya OR Lesotho OR Liberia OR Libya OR Madagascar OR Malawi OR Mali OR Mauritania OR Morocco OR Mozambique OR Namibia OR Niger OR Nigeria OR Rwanda OR "Sao Tome" OR Principe OR Senegal OR "Sierra Leone" OR Somalia OR Somaliland OR "South Africa" OR "South Sudan" OR Sudan OR Swaziland OR Tanzania OR Togo OR Tunisia OR Uganda OR Zambia OR Zimbabwe).mp. – ("South America" OR "Latin America" OR "Central America" OR Mexico OR Argentina OR Bolivia OR Brazil OR Chile OR Colombia OR Ecuador OR Guyana OR Paraguay OR Peru OR Suriname OR Uruguay OR Venezuela OR Belize OR "Costa Rica" OR "El Salvador" OR Guatemala OR Honduras OR Nicaragua OR Panama).mp – ("Middle East" OR "South‐East Asia" OR "Indian Ocean Island*" OR "South Asia" OR "Central Asia" OR Caucasus OR Afghanistan OR Azerbaijan OR Bangladesh OR Bhutan OR Burma OR Cambodia OR China OR Georgia OR India OR Iran OR Iraq OR Jordan OR Kazakhstan OR Korea OR "Kyrgyz Republic" OR Kyrgyzstan OR Lao OR Laos OR Lebanon OR Macao OR Mongolia OR Myanmar OR Nepal OR Oman OR Pakistan OR Russia OR "Russian Federation" OR "Saudi Arabia" OR Bahrain OR Indonesia OR Malaysia OR Philippines OR Sri Lanka OR Syria OR "Syrian Arab Republic" OR Tajikistan OR Thailand OR Timor‐Leste OR Timor OR Turkey OR Turkmenistan OR Uzbekistan OR Vietnam OR "West Bank" OR Gaza OR Yemen OR Comoros OR Maldives OR Mauritius OR Seychelles).mp – ("Pacific Islands" OR "American Samoa" OR Fiji OR Guam OR Kiribati OR "Marshall Islands" OR Micronesia OR New Caledonia OR "Northern Mariana Islands" OR Palau OR "Papua New Guinea" OR Samoa OR "Solomon Islands" OR Tonga OR Tuvalu OR Vanuatu).mp – ("Eastern Europe" OR Balkans OR Albania OR Armenia OR Belarus OR Bosnia OR Herzegovina OR Bulgaria OR Croatia OR Cyprus OR "Czech Republic" OR Estonia OR Greece OR "Isle of Man" OR Kosovo OR Latvia OR Lithuania OR Macedonia OR Malta OR Moldova OR Montenegro OR Poland OR Portugal OR Romania OR Serbia OR "Slovak Republic" OR Slovakia OR Slovenia OR Ukraine).mp
Systematic review key words	
– meta analysis/ – ((systematic* adj2 review*) or "meta‐analy*" or "meta analy*")
Evidence map key words	
– Evidence maps – evidence adj3 map – Evidence mapping – Evaluation map – Evaluation mapping – Systematic map – Systematic mapping – Descriptive mapping – Scientific uncertainties
Child well‐being key words	
– [(child abuse* OR child well‐being OR child protect* service* OR foster care OR child* adj3 abus* OR schoolchild*, adolescen*, teen*)]
Target age group key words	
– (young child* OR children OR pre‐schooler* OR pre‐schoolers*, kindergarten* OR kindergartener*, early child OR childhood, and early year OR years) – ("adolescen*" OR juvenile OR minors OR youth OR "young adult" OR "young women" OR "young men" OR girl* OR boy* OR (school adj6 student*) OR teen* OR schoolgirl* OR schoolboy*)

## ANNEX 3 AMSTAR‐2

**Table:**

S.no.	Question	Option
1.	Did the research questions and inclusion criteria for the review include the components of PICO?	Yes
		No
2.	Did the report of the review contain an explicit statement that the review methods were established prior to the conduct of the review and did the report justify any significant deviations from the protocol?	Yes
**		Partial Yes
		No
3.	Did the review authors explain their selection of the study designs for inclusion in the review?	Yes
		No
4.	Did the review authors use a comprehensive literature search strategy?	Yes
**		Partial Yes
		No
5.	Did the review authors perform study selection in duplicate?	Yes
		No
6.	Did the review authors perform data extraction in duplicate?	Yes
		No
7.	Did the review authors provide a list of excluded studies and justify the exclusions?	Yes
**		Partial Yes
		No
8.	Did the review authors describe the included studies in adequate detail?	Yes
		Partial Yes
		No
9.	Did the review authors use a satisfactory technique for assessing the risk of bias (RoB) in individual studies that were included in the review?	Yes
**		Partial Yes
		No
		Includes only NRSI/RCTs
10.	Did the review authors report on the sources of funding for the studies included in the review?	Yes
		No
11.	If meta‐analysis was performed did the review authors use appropriate methods for statistical combination of results?	Yes
**		No
		No meta‐analysis conducted
12.	If meta‐analysis was performed, did the review authors assess the potential impact of RoB in individual studies on the results of the meta‐analysis or other evidence synthesis?	Yes
		No
		No meta‐analysis conducted
13.	Did the review authors account for RoB in individual studies when interpreting/discussing the results of the review?	Yes
**		No
14.	Did the review authors provide a satisfactory explanation for, and discussion of, any heterogeneity observed in the results of the review?	Yes
		No
15.	If they performed quantitative synthesis did the review authors carry out an adequate investigation of publication bias (small study bias) and discuss its likely impact on the results of the review?	Yes
**		No
		No meta analysis conducted
16.	Did the review authors report any potential sources of conflict of interest, including any funding they received for conducting the review?	Yes
		No

## ANNEX 4 CODING TOOL

**Table:**

Coding variable	Example of information that may be recorded
Full references	Author, title, date of publication
Publication type	Academics, Journal, book, conference, thesis
Study Region	World Bank Region
Study Country	Name of the country
Status of study	Ongoing/completed
Data Type	Qualitative/Quantitative
Child categories	Target group (e.g., child/adolescent)*
Intervention	Type of intervention/domain included like Early childhood intervention categories.
Population filters	Orphans/vulnerable children etc.
Outcome	Type of outcome assessed like safety, health etc.
Study Quality	Low, medium, high
