# Supplementary material for: Mega‐map of systematic reviews and evidence and gap maps on the interventions to improve child well‐being in low‐ and middle‐income countries
Source: Campbell Syst Rev. 2020 Oct 28;16(4):e1116. doi: 10.1002/cl2.1116 (PMC8356294; doi:10.1002/cl2.1116)
Supplement: Supplementary file 1 — Supporting information [file CL2-16-e1116-s001.docx]

**Online supplements**

***List of online supplementary materials***

Link to online interactive EGM: To be added

(snap shot of the Mega map added)


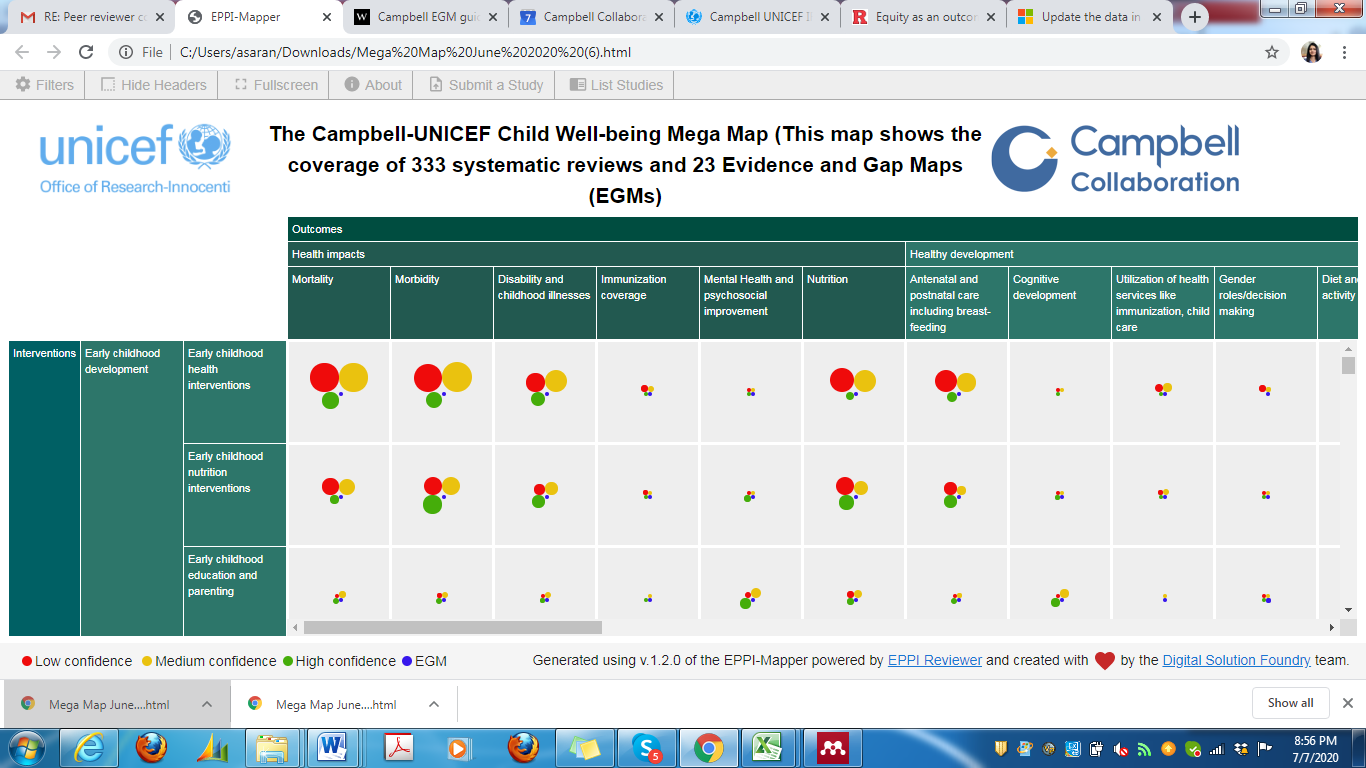


Links to a series of five briefs, which provide an overview of available evidence shown in the Campbell -UNICEF Mega map of the effectiveness of interventions to improve child welfare in low- and middle-income countries (LMICs):

1. Evidence and gap map research brief: UNICEF Strategic Plan 2018–2021 Goal Area 1: Every Child Survives and Thrives

<https://www.unicef-irc.org/publications/1068-evidence-and-gap-map-research-brief-unicef-strategic-plan-2018-21-goal-area-1.html>

1. Evidence and gap map research brief: UNICEF Strategic Plan 2018–2021 Goal Area 2: Every Child Learns

<https://www.unicef-irc.org/publications/1069-evidence-and-gap-map-research-brief-unicef-strategic-plan-2018-21-goal-area-2.html>

1. Evidence and gap map research brief: UNICEF Strategic Plan 2018–2021 Goal Area 3: Every Child is Protected from Violence and Exploitation

<https://www.unicef-irc.org/publications/1070-evidence-and-gap-map-research-brief-unicef-strategic-plan-2018-21-goal-area-3.html>

1. Evidence and gap map research brief: UNICEF Strategic Plan 2018–2021 Goal Area 4: Every Child Lives in a Safe and Clean Environment

<https://www.unicef-irc.org/publications/1071-evidence-and-gap-map-research-brief-unicef-strategic-plam-2018-21-goal-area-4.html>

1. Evidence and gap map research brief: UNICEF Strategic Plan 2018–2021 Goal Area 5: Every Child has an Equitable Chance in Life

<https://www.unicef-irc.org/publications/1072-evidence-and-gap-map-research-brief-unicef-strategic-plan-2018-21-goal-area-5.html>
